# Performance of the coronary calcium score in an outpatient chest pain clinic and strategies for risk stratification

**DOI:** 10.1002/clc.23539

**Published:** 2021-01-12

**Authors:** Weiting Huang, Leon Ming Hsien Lim, Amirzeb S/O. Aurangzeb, Cheney Jianlin Wong, Natalie Si Ya Koh, Zijuan Huang, Hooi Khee Teo, Terrance Siang Jin Chua, Swee Yaw Tan

**Affiliations:** ^1^ Cardiology, National Heart Centre Singapore 5 Hospital Drive Singapore 169609 Singapore; ^2^ Yong Loo Lin School of Medicine 10 Medical Drive Singapore 117597 Singapore

**Keywords:** chest pain, coronary artery calcium, coronary artery disease, risk score

## Abstract

**Background:**

Coronary artery calcium score (CAC) is an objective marker of atherosclerosis. The primary aim is to assess CAC as a risk classifier in stable coronary artery disease (CAD).

**Hypothesis:**

CAC improves CAD risk prediction, compared to conventional risk scoring, even in the absence of cardiovascular risk factor inputs.

**Methods:**

Outpatients presenting to a cardiology clinic (*n* = 3518) were divided into two cohorts: derivation (*n* = 2344 patients) and validation (*n* = 1174 patients). Adding logarithmic transformation of CAC, we built two logistic regression models: Model 1 with chest pain history and risk factors and Model 2 including chest pain history only without risk factors simulating patients with undiagnosed comorbidities. The CAD I Consortium Score (CCS) was the conventional reference risk score used. The primary outcome was the presence of coronary artery disease defined as any epicardial artery stenosis≥50% on CT coronary angiogram.

**Results:**

Area under curve (AUC) of CCS in our validation cohort was 0.80. The AUC of Models 1 and 2 were significantly improved at 0.88 (95%CI 0.86–0.91) and 0.87 (95%CI 0.84–0.90), respectively. Integrated discriminant improvement was >15% for both models. At a pre‐specified cut‐off of ≤10% for excluding coronary artery disease, the sensitivity and specificity were 89.3% and 74.7% for Model 1, and 88.1% and 71.8% for Model 2.

**Conclusion:**

CAC helps improve risk classification in patients with chest pain, even in the absence of prior risk factor screening.

## 1 INTRODUCTION

Chest pain is one of the top referral reasons to cardiology outpatient clinics. The prevalence of coronary artery disease at outpatient chest pain clinics varies around 10–20%.^1^, ^2^, ^3^ While majority of patients are told to be free of obstructive coronary artery disease (CAD) after examination and testing, this clinical encounter is a good opportunity to provide advice and initiate therapy for primary prevention.

The coronary artery calcium (CAC) test has rapid turnaround and requires little preparation. This provides the option of same‐day testing at an outpatient clinic. Depending on chest pain history, low risk patients with low CAC may be discharged on the same day with chest pain advice, saving future visits for cardiac tests and follow up. A zero calcium score is strong negative risk predictor for coronary artery disease, with a negative predictive value of 99%, sensitivity of 91%, and specificity of 64%.^4^

Moreover, conventional risk scores, such as the CAD Consortium Score (CCS), depend on prior knowledge of personal cardiovascular risk factors. This disadvantages patients who do not have previous health screening. The CAC, which reflects arterial age under the influence of underlying comorbidities, can be helpful when cardiovascular risk factors are not available. In the setting stable angina where plaque formation and remodeling occur over years, underlying comorbidities leave a physiological imprint of accelerated coronary atherosclerosis, which can be quantified by the CAC.

The CAC also guides prescription of statin therapy,^5^, ^6^ which is important in the primary prevention of myocardial infarction. While functional tests such as myocardial perfusion imaging and stress echocardiogram give information on the area and extent of ischemia due to a coronary artery lesion, the CAC gives physicians actionable information to modify risk through optimal medical therapy. Statins are favored in non‐zero calcium scores, especially in those aged 55 and above.^7^

The primary aim of this study was to determine the performance CAC as a risk classifier when added to conventional risk factor variables and chest pain history, by creating a risk model that can be applied to daily clinical use. One common challenge in clinic is lack of prior health screening, leading to underreporting of pre‐existing cardiovascular risk factors. Hence, we also looked at the performance of the CAC in the absence of cardiovascular risk factor variables.

## 2 METHODS

This was a cross sectional study. Recruitment occurred between January 01, 2014 and December 31, 2017. Recruitment occurred at the National Heart Centre Singapore cardiology outpatient clinics. Consecutive patients with chest pain referred to the CT laboratory for testing during that period were recruited. The National Heart Centre Singapore is a 185‐bed national and regional referral centre for cardiovascular medicine.

All procedures followed were in accordance with the ethical standards of the responsible committee on human experimentation (institutional and national) and with the Helsinki Declaration. Informed consent was obtained from all patients for being included in the study.

The total sample size was 3518 patients. All patients underwent computed tomography coronary angiogram (CTCA) with readouts of degree of coronary artery stenosis and CAC.

The primary outcome of interest was the presence of obstructive coronary artery disease, defined as any epicardial artery stenosis of ≥50% on CT coronary angiogram. Epicardial artery stenosis of ≥50% was the same outcome used by the CAD Consortium score, our reference risk score.^8^

All patients presenting to the CT laboratory had their baseline demographics, chest pain history and self‐reported cardiovascular risk factors collected by a nurse clinician. The CAD I consortium score^9^ was the conventional risk calculator used for comparison.

The CTCA and CAC were performed using a Toshiba Aquilion ONE scanner with 160 mm coverage and 320 slice detector. Calcium scan was prospectively gated and scanned over a single heartbeat with a gantry rotation and X‐ray exposure time of 0.35 s, 0.5 mm slice collimation, tube voltage of 120 kV, and tube current of 140 mA. Images were reconstructed at 3.0 mm slice thickness for calcium score. Assessment was carried out using the Vitrea Calcium software and Agatston scoring schema. All studies were assessed for arterial lumen stenosis for all coronary arterial segments. Images were assessed using volume‐rendered images, curved multiplanar reformations, and cross‐sectional images in available phases as well as from sharp and standard kernels. Visual assessment of arterial segment lumen diameter stenosis was carried out. In assessing stenosis, the minimum lumen diameter was identified for each arterial segment and then compared with a reference site of a disease‐free site in closest proximity to the lesion site.

The outcome of significant coronary artery stenosis on CTCA was determined by two independent radiologists who were not involved in the baseline data collection. Disagreements between the two readers was resolved by consensus.

The study complied with the Declaration of Helsinki and was approved by our Centre's Institutional Review Board.

### 2.1 Statistical methods

Continuous normally distributed variables were compared by t‐test and categorical variables by chi square test in univariate analysis. Normally distributed variables were presented as mean and standard deviation, while non‐normal variables were presented as median and its interquartile range.

The cohort was divided into derivation and validation cohorts, in two thirds (2344 patients) and one third (1174 patients), respectively. We built two logistic regression models including calcium score to predict the probability of having obstructive coronary artery disease: Model 1 (traditional risk factors, chest pain history, logarithmic‐transformed calcium score^10^) and Model 2 (chest pain history and logarithmic‐transformed calcium score, omitting self‐reported cardiovascular risk factors inputs of diabetes mellitus, hypertension and dyslipidemia), simulating patients who do not know their underlying cardiovascular risk factors at the initial clinic visit. Only variables with *p*‐value >.1 in univariate analyses were considered for regression model building. The logistic regression models were then externally validated in the validation cohort.

These models were then compared to the previously published CAD I consortium^9^ score (CCS) which uses cardiovascular risk factors and the Diamond‐Forrester chest pain history for risk prediction. We calibrated the CCS using our derivation cohort.

Discriminative abilities of the different models were evaluated by the area under to receiver operating curve, net reclassification index (NRI) and integrated discrimination improvement (IDI)^11^ in the validation cohort. Goodness‐of‐fit was assessed by the the Hosmer‐Lemeshow test. Internal validation in the derivation cohort was further tested by K‐fold cross validation.

All statistical analysis was performed on Stata Version 14.0 (StataCorp. 2015. College Station, TX: StataCorp LP). A *p*‐value of <.05 was considered statistically significant.

## 3 RESULTS

The baseline demographics of the cohort (presented separately as the derivation and validation groups) are presented in Table 1. The derivation and validation cohorts were comparable, as shown by non‐significant *p*‐values, except for average age where the derivation cohort was slightly older than the validation cohort (53.59 years vs. 52.94 years),. The age range was 17–80 years old, with a mean of 54 years old. The prevalence of significant coronary artery disease in the cohort was 15%. The cohorts were intermediate risk patients, with mean CCS probabilities of 14.9% and 14.3% in the derivation and validation cohorts respectively.

**TABLE 1:** Demographics of cohort

	Derivation cohort			Validation cohort			
	All patients (*n* = 2344)	Presence of CAD (*n* = 350)	^a^ *p*‐value	All patients (*n* = 1174)	Presence of CAD (*n* = 176)	^a^ *p*‐value	^b^ *p*‐value
Age (years)	53.59 ± 12.38	61.10 ± 12.30	<.001	52.94 ± 12.74	58.76 ± 10.41	<0.001	0.029
Race							
Chinese	1743 (74.3%)	271 (77.4%)	1.0	862 (73.4%)	136 (77.3%)	1.0	1.0
Malay	150 (6.4%)	36 (10.2%)	.748	127 (10.8%)	15 (8.5%)	.248	.662
Indian	244 (10.4%)	18 (5.1%)	.248	81 (6.9%)	10 (5.7%)	.416	.542
Others	208 (8.8%)	25 (7.1%)	.190	104 (8.8%)	15 (8.5%)	.720	.901
Male	1306 (55.7%)	238 (68.0%)	<.001	681 (58.0%)	126 (76.6%)	<.001	.196
Hypertension	414 (17.6%)	145 (41.4%)	<.001	197 (16.7%)	75 (42.6%)	<.001	.515
Diabetes Mellitus	166 (7.1%)	55 (15.7%)	<.001	70 (5.9%)	29 (16.5%)	<.001	.211
Dyslipidemia	739 (31.5%)	227 (64.8%)	<.001	363 (30.9%)	109 (61.9%)	<.001	.714
Family history of premature CAD	616 (26.1%)	129 (36.8%)	<.001	338 (28.7%)	66 (37.5%)	.006	.102
History of Smoking	399 (17.0%)	76 (21.7%)	.012	210 (17.8%)	53 (30.1%)	<.002	.522
Calcium score	1 [0,88]	277 [103, 650]	<.001	2 [0, 78]	239 [65, 548]	<.001	.182
Zero calcium score	1082 (48.0%)	21 (6.3%)	<.001	527 (47.0%)	10 (5.9%)	<.001	.284
Chest pain history							
Typical	1237 (52.7%)	219 (62.5%)	<.001	618 (52.6%)	117 (66.4%)	<.001	.906
Atypical	1016 (43.3%)	97 (27.7%)	<.001	512 (43.6%)	44 (25.0%)	.017	.863
Non‐anginal	91 (3.8%)	34 (9.7%)	Ref	44 (3.7%)	15 (8.5%)	Ref	1.0
Probability of coronary artery disease by CAD consortium score (%)	8.73 (4.08, 19.24)	27.26 (14.10, 45.39)	<.001	8.17 (3.92, 19.07)	25.34 (13.45, 39.77)	<.001	.282

Abbreviation: CAD, coronary artery disease.

^a^ Indicates p‐value comparisons between patients with obstructive coronary artery disease and patients without.

^b^ Indicates p‐value comparisons between derivation and validation cohorts.

### 3.1 Performance of a CAC≥1 in predicting coronary artery disease in study cohort

A calcium score of zero suggests minimal coronary atherosclerosis. Forty‐eight percent (1609/3372) of our cohort with chest pain had zero calcium score. The performance CAC≥1 in predicting obstructive coronary artery disease was: sensitivity 93.8%, specificity 54.9%, and negative predictive value 98.1%. The performance of CAC at previously established cut‐offs^12^ in our clinical cohort is shown in Supplementary Table S1.

### 3.2 Performance of risk models consisting of logarithmic transformed calcium score, with and without cardiovascular risk factors

In view of the common challenge of lack of prior cardiovascular risk factor screening, we created two predictive models, one including the component of cardiovascular risk factors (Model 1) and the other without (Model 2).

For Model 1, apart from the chest pain history, variables used in modeling included age, gender, hypertension, diabetes mellitus, dyslipidemia, family history of early coronary artery disease (defined as first‐degree relative with cardiovascular event at age < 55 years in males and age < 65 years in females) and logarithmic transformed CAC. For Model 2, the variables included were chest pain history, age, gender, family history of early coronary artery disease, and logarithmic transformed CAC. (Table 2).

**TABLE 2:** Predictive model building using derivation cohort (*n* = 2344)

	Univariate logistic regression		Multivariate logistic regression					
			Model 1 with risk factor variables			Model 1 without risk factor variables		
	Odds ratio ± SE	*p*‐value	Odds ratio ± SE	Beta coefficient	*p*‐value	Odds ratio ± SE	Beta coefficient	p‐value
Age (years)	1.06 ± 0.01	<.001	0.99 ± 0.01	−0.002 ± 0.008	0.747	1.01 ± 0.01	0.008 ± 0.008	0.316
Race								
Chinese (Ref)	1.0							
Malay	0.74 ± 0.19	.248						
Indian	0.94 ± 0.18	.748						
Others	0.74 ± 0.16	.190						
Male	1.84 ± 0.22	<.001	1.66 ± 0.25	0.44 ± 0.16	.007	1.46 ± 0.23	0.37 ± 0.15	0.017
Hypertension	4.53 ± 0.11	<.001	1.51 ± 0.28	0.41 ± 0.18	.024			
Diabetes Mellitus	3.16 ± 0.55	<.001	1.15 ± 0.28	0.14 ± 0.24	.561			
Dyslipidemia	5.34 ± 0.65	<.001	2.85 ± 0.51	1.05 ± 0.17	<.001			
Family history of premature coronary artery disease	1.81 ± 0.22	<.001	1.25 ± 0.21	0.22 ± 0.16	.180	1.79 ± 0.28	0.58 ± 0.15	<0.001
History of Smoking	1.43 ± 0.20	.112						
Log (calcium score+1)	0.63 ± 0.03	<.001	1.84 ± 0.07	0.61 ± 0.05	<.001	1.85 ± 0.07	0.61 ± 0.03	<0.001
Calcium score groups								
0	Reference							
1–99	5.32 ± 1.37	<0.001						
100‐400(Ref)	30.31 ± 7.52	<.001						
401 and above	72.59 ± 18.93	<.001						
Chest pain history								
Typical	2.77 ± 0.63	<.001	2.50 ± 0.83	0.91 ± 0.33	.006	2.74 ± 0.88	1.01 ± 0.32	<0.001
Atypical	0.49 ± 0.06	<.001	0.57 ± 0.09	−0.54 ± 0.16	.001	0.43 ± 0.06	−0.58 ± 0.15	0.002
Non‐anginal (Ref)	1.0		1.0	Ref		1.0	Ref	
Model Constant			0.01 ± 0.00	−4.54 ± 0.51	<.001	0.01 ± 0.00	−4.59 ± 0.49	<0.001
Area under the curve			0.893 ± 0.009			0.880 ± 0.010		
Adjusted R‐square			0.367			0.330		

K‐fold cross internal validations (five‐fold) were performed in the derivation cohort on both models. Root mean square error for Model 1 was 0.269–0.294 with *r2* of 0.282–0.430; root mean square error for Model 2 without cardiovascular risk factors was 0.275–0.304 with *r2* of 0.303–0.356.

**FIGURE 1:**
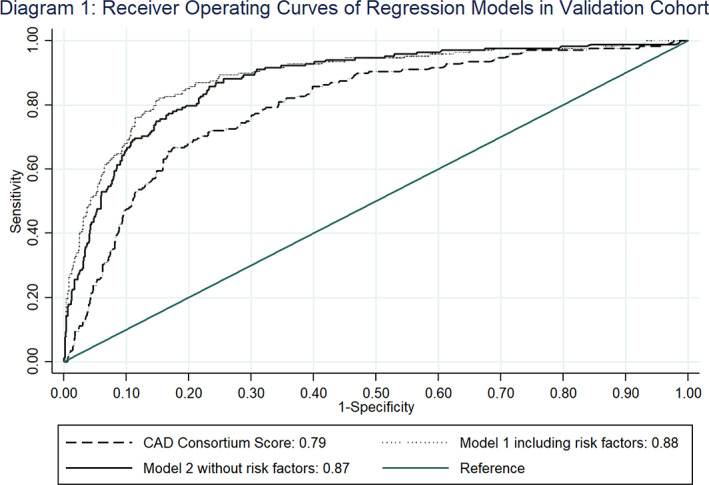
AUC curves comparing new risk scores and traditional CAD I Consortium Score

Receiver operator curves (ROC) of the calibrated CCS and our two models in the validation cohort were: CCS 0.798 (95%CI 0.762–0.834); Model 1 0.889 (95%CI 0.860–0.918); Model 2 (without cardiovascular risk factors) 0.875 (95%CI 0.846–0.904). Both Models 1 and 2 performed significantly better than CCS *p*‐value<.001 and Model 1 performed better than Model 2 *p*‐value = .009 (see Figure 1). All models were of good fit, by non‐significant Hosmer‐Lemeshow tests.

The NRI of Model 1 and Model 2 over conventional CCS were: Model 1 continuous event NRI 0.465 (95% CI 0.412–0.526), continuous non‐event NRI 0.683 (95%CI 0.660–0.713) and Model 2 continuous event NRI 0.388 (95%CI 0.329–0.463), continuous non‐event NRI 0.658 (95%CI 0.634–0.683). The IDI over CCS were 0.215 (95%CI 0.181–0.248) and 0.177 (95%CI 0.146–0.211) for Model 1 and Model 2, respectively. Table 3 shows the improvement in classification of Model 1 over CCS, at pre‐specified cut‐offs.

**TABLE 3:** Reclassification improvement of Model 1 with reference to the CAD Consortium Score

Risk category by calibrated CAD Consortium Score	Risk categories by Model 1				
	Obstructive coronary artery disease present on CT coronary angiogram				
	<10%	10% to <20%	20% to <30%	30% and above	Total
<10%	32 (40.0%)	20 (25.0%)^a^	13 (16.3%)^a^	15 (18.75%)^a^	80 (16.1%)
10% to <20%	18 (16.7%)	13 (12.0%)	22 (20.4%)^a^	55 (50.9%) ^a^	108 (21.7%)
20% to <30%	5 (5.2%)	8 (8.3%)	6 (6.3%)	77 (80.2%)^a^	96 (19.3%)
30% and above	5 (2.4%)	5 (2.4%)	8 (3.8%)	195 (91.5%)	214 (42.9%)
Total	69 (12.1%)	46 (9.3%)	49 (9.9%)	342 (68.9%)	497 (100%)
	Obstructive coronary artery disease absent on CT coronary angiogram				
	<10%	10% to <20%	20% to <30%	30% and above	Total
<10%	1523 (87.5%)	133 (7.7%)	56 (3.2%)	27 (1.6%)	1739 (60.5%)
10% to <20%	422 (65.7%)^a^	107 (16.7%)	53 (8.3%)	60 (9.4%)	642 (22.3%)
20% to <30%	116 (49.4%)^a^	41 (17.5%)^a^	24 (10.2%)	54 (23.0%)	235 (8.2%)
30% and above	75 (29.0%)^a^	24 (9.3%)^a^	24 (9.3%)^a^	136 (52.5%)	259 (9.0%)
Total	2136 (74.3%)	305 (10.6%)	157 (5.5%)	277 (9.6%)	2875 (100%)

^a^ Represents groups of patients who were appropriately uptriaged or downtriaged by addition of calcium score.

Using a probability cut‐off of ≤10% as a gatekeeper for no further testing, the sensitivity, specificity, and negative predictive value for Model 1 in the validation cohort were 89.3%, 74.7% and 97.5% respectively. Approximately 65% of our validation cohort had a risk probability of ≤10% using Model 1. In Model 2 where cardiovascular risk factors were not included, the sensitivity, specificity and negative predictive value at the same cut‐off were 88.1%, 71.8% and 97.2%. This contrasted with the conventional CCS where similar cut‐offs had lower sensitivity, specificity, and negative predictive value at 85.8%, 61.3% and 95.5%. The findings suggested that CAC was a safety net, even when knowledge of prior risk factors was not available.

The performance of CCS, Model 1 and Model 2 at pre‐specified cut‐offs up to 30% are presented in Table 4. Logarithmic transformed calcium score, chest pain history and dyslipidemia were the three most important variables in predicting presence of coronary artery disease in our patient cohort. Details on the importance of other features are shown in Supplementary Figure S2.

**TABLE 4:** Performance of Model 1, Model 2 and CAD Consortium Score at pre‐specified cut‐offs in validation vohort

Cut‐off for predicting presence of coronary artery disease	0.10	0.20	0.30
Performance of Model 1 with calcium score including risk factors			
Sensitivity	89.3%	82.1%	67.3%
Specificity	74.7%	84.3%	90.3%
Negative predictive value	97.5%	96.4%	94.0%
Area under curve	0.82	0.83	0.79
Proportion in validation cohort above cut‐off	34.8%	20.3%	18.2%
False positive rate	0.25	0.15	0.09
False negative rate	0.10	0.17	0.32
Performance of Model 2 with calcium score without risk factors			
Sensitivity	88.1%	78.0%	68.5%
Specificity	71.8%	82.4%	89.1%
Negative predictive value	97.2%	95.5%	94.1%
Area under curve	0.80	0.80	0.79
Proportion in validation cohort above cut‐off (%)	37.1%	26.6%	19.5%
False positive rate	0.28	0.17	0.10
False negative rate	0.11	0.22	0.31
Performance of CAD Consortium Score			
Sensitivity	85.8%	64.2%	39.2%
Specificity	61.3%	83.8%	92.0%
Negative predictive value	96.1%	93.0%	89.6%
Area under curve	0.74	0.74	0.66
Proportion in validation vohort above cut‐off	45.7%	23.4%	12.6%
False positive rate	0.38	0.16	0.08
False negative rate	0.14	0.35	0.60

## 4 DISCUSSION

Our study showed that by incorporating calcium score into the risk prediction model, the performance of our model is comparable to functional cardiac testing, which is one of the recommended strategies by the ACC/AHA^13^ for investigation of chest pain syndromes. Using the pre‐specified cut‐off of ≤10% as gatekeeper for no further testing, the sensitivity and specificity for Model 1 (inclusive of cardiovascular risk factors) were 89.3% and 74.7% and Model 2 (without cardiovascular risk factors) 88.1% and 71.8%, respectively. Functional stress tests to diagnose coronary artery disease such as stress echocardiography and myocardial perfusion imaging tests have comparable sensitivities and specificities of 87% and 72%, and 83% and 77%, respectively.^14^ Approximately 65% of our study population had risk scores <10% after application of the calcium score. This suggests that majority of patients may be discharged at the same clinic visit after a CT calcium score. This can impact workflow and reduce follow‐up clinic visits.

Additionally, a zero calcium score confers good prognosis in the setting of chest pain with 1% annual event rate,^12^ despite the risk of having non‐calcified plaques.^15^ The annual event rate of a zero calcium score is lower than a negative stress test (2.1%), based on the PROMISE study; majority of cardiac events occurred in patients with non‐zero calcium scores.^12^ This shows the additional prognostic value of zero calcium score. A non‐zero calcium score hence provides opportunity for initiation of statins, especially in those aged 55 and above or with an increased Atherosclerotic Cardiovascular Disease (ASCVD) score, as recommended by the 2018 ACC/AHA Cholesterol Guidelines.^7^ Apart from diagnosing obstructive coronary artery disease, primary prevention advice is essential for cardiovascular disease management. For every 1 mmol/dl LDL‐C reduction with statin therapy, the relative risk of major adverse cardiovascular events is reduced by approximately 20–25%, and all‐cause mortality is reduced by 10%.^16^ The CAC provides actionable information that may not be available from a negative stress imaging test. The rates of significant plaque in the setting of zero calcium in our study is 1.9%, which is largely similar to findings from other chest pain studies.^12^, ^17^, ^18^

Conversely, patients with risk scores 30% and above for CCS, Model 1 and 2, or CAC > 400 have very high likelihood of coronary artery disease (see Table 4). At the cut‐off of >0.30, the specificities of Model 1 and 2 were approximately 90%; at cut‐off CAC > 400, the specificity in detecting coronary artery disease was 95.3%. Hence, physicians may consider initiating aspirin and statins at the same clinic visit while arranging for invasive coronary angiogram or functional imaging tests, with early follow up to ensure stability of symptoms. For patients in risk regions of >10% functional stress test such as treadmill exercise echocardiogram should be performed to assess ischemia in the presence of atherosclerosis, for prognostication.

The cost of CT coronary angiogram at our institution is $1304; while that of CAC is $500. Thirty‐five percent of our study population had risk scores >10% requiring further testing, and a stress echocardiogram costs $772. As such, this strategy can save up to $800 in low risk patients; even if patient undergoes the functional stress test after the CAC, the cost will be similar to a CTCA. Additionally, studies have shown that CTCA may lead to increased invasive coronary angiography,^19^ and additional functional testing may have to be performed for prognostication, and to defer invasive coronary angiography.^20^

The advantage of the CT calcium score is the ease of incorporating a same‐day CAC test at the outpatient setting. Preparation for the test is minimal unlike other cardiovascular stress test which requires cessation of certain medications^21^ or abstinence from caffeinated products.^22^ It also does not require additional heart rate lowering agents^23^ or blood tests to ensure adequate renal function. The radiation dose of the CT calcium score in our laboratory is low at 0.5 mSV compared to a full CT coronary angiogram (2.7 mSV)^24^ and myocardial perfusion imaging test (5.3 mSV).^25^

There are limitations to our study. The study population was intermediate risk, with average CAD consortium score of 15%. These patients were referred from the primary care setting to the cardiology specialist clinic for investigation of chest pain. The risk profile of the study population was lower compared to previous cardiac CT‐based studies,^17^, ^19^, ^26^ hence further calibration may be required before the model can be used on higher risk populations. The study cohort was a multi‐ethnic Southeast Asian population; the lower cardiovascular risk profile of Asians (apart from South Asians) when compared to Western populations may contribute to the lower risk profiles seen in our study. The reference primary outcome was CT coronary angiogram detected stenosis, instead of gold standard invasive coronary angiography. Long term follow‐up for major adverse cardiovascular outcomes was also not available.

Our study adds to literature by addressing the common challenge of unknown cardiovascular risk factors at the first clinic visit. As CAC is reflective of the arterial age^27^ under the influence of cardiovascular comorbidities such as diabetes mellitus, the study showed that the CAC is a good risk classifier, even in the absence of such information. Without knowledge of cardiovascular risk factors, the ROC performance of Model 2 remains good at 0.87 in the validation cohort.

We also supported findings from prior studies; prior studies^15^, ^28^ previously discussed the value of a zero calcium score in chest pain clinics and previous work from Genders et al^9^ added calcium score for risk prediction for chest pain patients, similar to our Model 1. We extrapolated these findings to our cohort and performed both internal and external validation. The ROC on our validation cohort of Model 1 and Model 2 compares favorably to the model incorporating CAC by Genders et al, where their ROC ranges 0.78 to 0.81.

## 5 CONCLUSION

The coronary CAC is a useful aid to streamline workflow in chest pain clinics at an outpatient setting.

## CONFLICT OF INTEREST

The authors declare no potential conflict of interest.

## Supporting information


**Table S1:** Performance of Calcium Score at a priori determined calcium categories
**Supplementary Figure S2**: Normogram showing Feature Importance in Predicting Presence of coronary artery disease

## Data Availability

The data that support the findings of this study are available from the corresponding author, W Huang upon reasonable request.
