# Supplementary material for: Performance of the coronary calcium score in an outpatient chest pain clinic and strategies for risk stratification
Source: Clin Cardiol. 2021 Jan 12;44(2):267–75. doi: 10.1002/clc.23539 (PMC7852173; doi:10.1002/clc.23539)

**Supplement**

**Supplementary Table S1:**

| **Table S1: Performance of Calcium Score at a priori determined calcium categories** | | | |
| --- | --- | --- | --- |
| **Cut-off for predicting presence of coronary artery disease** | >0 | >99 | >400 |
| Sensitivity | 93.8% | 73.2% | 36.4% |
| Specificity | 54.9% | 84.8% | 95.3% |
| Negative Predictive Value | 98.1% | 94.8% | 89.7% |
| Area Under Curve | 0.74 | 0.79 | 0.66 |
| Proportion in Validation Cohort above cut-off | 52.2% | 23.7% | 9.3% |
| False Positive rate | 0.38 | 0.15 | 0.04 |
| False Negative rate | 0.06 | 0.26 | 0.63 |

**Supplementary Figure S2: Normogram showing Feature Importance in Predicting Presence of coronary artery disease**


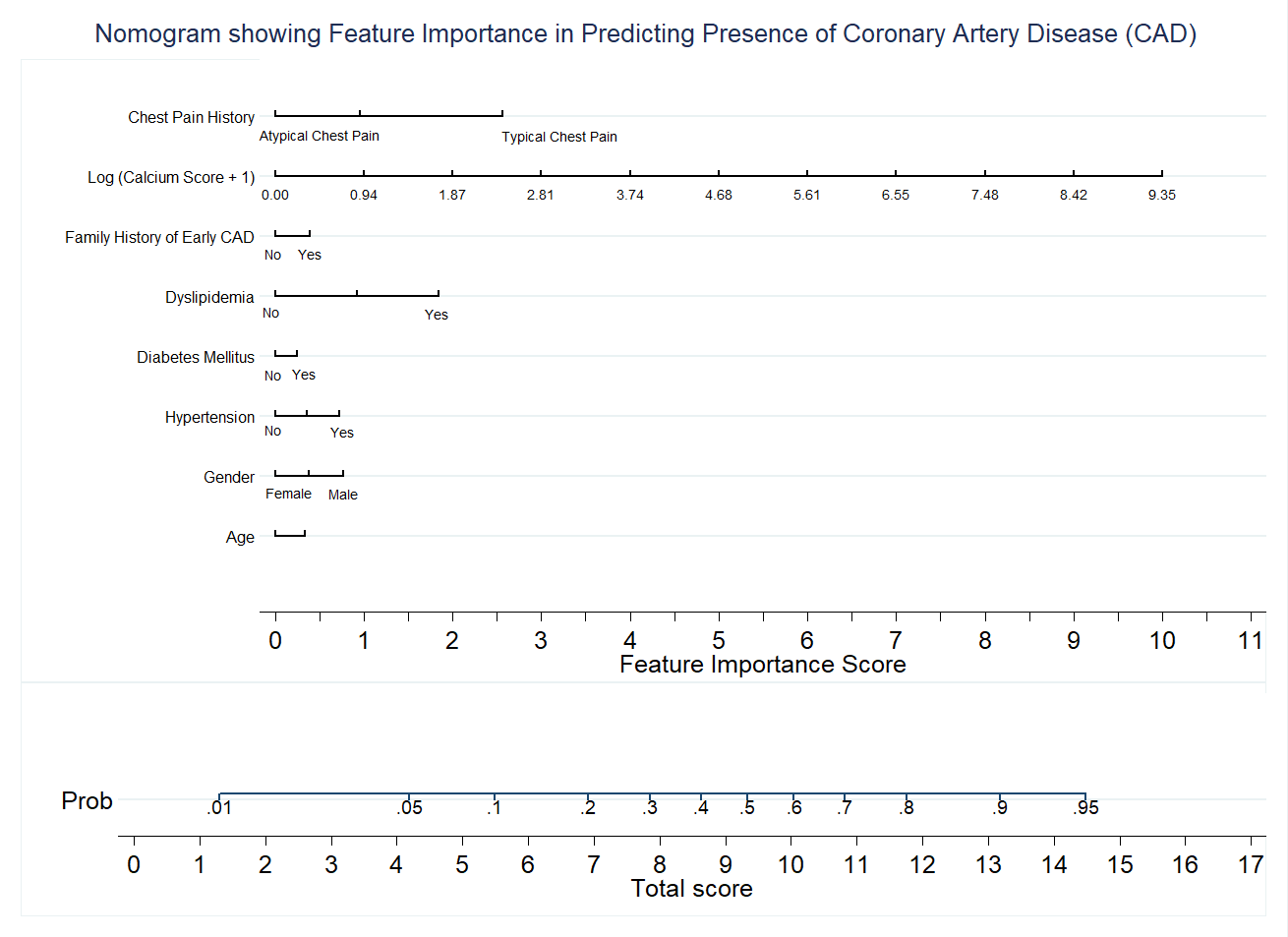

Supplement: Supplementary file 1 — Table S1: Performance of Calcium Score at a priori determined calcium categories Supplementary Figure S2: Normogram showing Feature Importance in Predicting Presence of coronary artery disease [file CLC-44-267-s001.docx]
